# Errors in Key Points, Abstract, Results, Table 2, and Conflict of Interest Disclosures

**DOI:** 10.1001/jamanetworkopen.2025.10736

**Published:** 2025-04-10

In the Original Investigation titled “Implementing Accuracy, Completeness, and Traceability for Data Reliability,”^1^ published March 10, 2025, the respective percentage values in Key Points given as 46.7% and 95.6% should have been 46.1% and 96.6%. In the Abstract Results, main article Results, and Table 2, the upper bound of the 95% CI given as 1.1% for advanced completeness should have been 107.4%. In the Conflict of Interest Disclosure for Dr Bradbury, the phrase “other from Verantos Inc during the conduct of the study” should have been removed. This article was corrected.^1^
